# 4-Decyl­phenyl 4-benz­yloxy-3-methyl­benzoate

**DOI:** 10.1107/S1600536810028965

**Published:** 2010-07-24

**Authors:** H. K. Arun Kashi, B. S. Palakshamurthy, M. VinduVahini, H. T. Srinivasa, H. C. Devarajegowda

**Affiliations:** a Department of Physics, Yuvaraja’s College (Constituent College), University of Mysore, Mysore 570 005, Karnataka, India; b Department of Physics, Sri D Devaraja Urs Govt. First Grade College, Hunsur 571 105, Mysore District, Karnataka, India; c Raman Research Institute, C. V. Raman Avenue, Sadashivanagar, Bangalore, Karnataka, India

## Abstract

In the title compound, C_31_H_38_O_3_, the central benzene ring makes dihedral angles of 66.06 (9) and 65.21 (8)°, respectively, with the benzyl and 4-decyl­phenyl rings.

## Related literature

For general background to benzyl­oxybenzoate, see: Laschat (2009 ▶); Meter & Klanderman (1973 ▶); Young *et al.* (1974 ▶); Tinn *et al.* (1982 ▶). For the synthesis, see: Sadashiva & Subba (1975 ▶); Sadashiva (1979 ▶); Hari *et al.* (2009 ▶). For related structures, see: Blake *et al.* (1996 ▶); Chin & Goodby (1986 ▶).
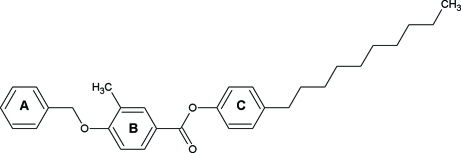


## Experimental

### 

#### Crystal data

C_31_H_38_O_3_
*M* *_r_* = 458.61
Triclinic, 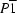

*a* = 9.3684 (16) Å
*b* = 11.168 (2) Å
*c* = 15.204 (3) Å
α = 68.588 (11)°
β = 87.274 (11)°
γ = 65.578 (10)°
*V* = 1338.3 (4) Å^3^
*Z* = 2
Mo *K*α radiation
μ = 0.07 mm^−1^
*T* = 295 K
0.22 × 0.15 × 0.12 mm

#### Data collection

Bruker SMART CCD area-detector diffractometer
Absorption correction: ψ scan (*SADABS*; Sheldrick, 2004 ▶) *T*_min_ = 0.987, *T*_max_ = 0.992
23986 measured reflections
6737 independent reflections
3840 reflections with *I* > 2σ(*I*)
*R*_int_ = 0.034

#### Refinement

*R*[*F* ^2^ > 2σ(*F* ^2^)] = 0.053
*wR*(*F* ^2^) = 0.183
*S* = 1.04
6737 reflections
308 parameters
6 restraints
H-atom parameters constrained
Δρ_max_ = 0.37 e Å^−3^
Δρ_min_ = −0.28 e Å^−3^

### 

Data collection: *SMART* (Bruker, 2001 ▶); cell refinement: *SAINT* (Bruker, 2001 ▶); data reduction: *SAINT*; program(s) used to solve structure: *SHELXS97* (Sheldrick, 2008 ▶); program(s) used to refine structure: *SHELXL97* (Sheldrick, 2008 ▶); molecular graphics: *ORTEP-3* (Farrugia, 1997 ▶); software used to prepare material for publication: *SHELXL97*.

## Supplementary Material

Crystal structure: contains datablocks I, global. DOI: 10.1107/S1600536810028965/gw2084sup1.cif

Structure factors: contains datablocks I. DOI: 10.1107/S1600536810028965/gw2084Isup2.hkl

Additional supplementary materials:  crystallographic information; 3D view; checkCIF report

## Figures and Tables

**Fig. 1.**
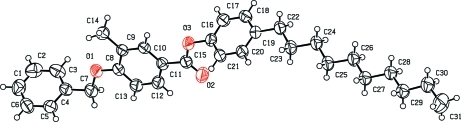
The asymmetric unit of the title compound. Displacement ellipsoids are drawn at the 50% probability level.

**Fig. 2.**
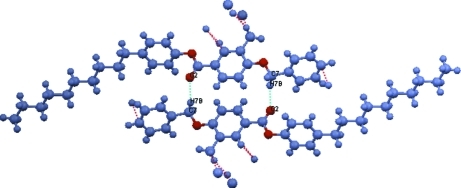
Packing of the molecules with dotted line weak intermolecular hydrogen bond.

## supplementary crystallographic information

### Comment

Liquid crystals are unique functional soft materials that possess both order and mobility at the molecular and supramolecular level.One of the major issues in liquid crystal research today is still the poor knowledge of structure-property relationships and thus the synthesis of whole series of structurally related compounds is required in order to allow the design of liquid crystalline and other physical properties (Laschat, 2009) Several benzyloxy derivative liquid crystals were reported with terminal alkyl and alkoxy chains (Meter *et al.*, 1973), and these compounds were shown to be of nematic mesophases (Young *et al.*, 1974; Tinn *et al.*, 1982). Terminal carbonitrile group containing liquid crystals were also synthesized and studied for their positive dielectric anisotropy (Sadashiva *et al.*, 1975; Sadashiva, 1979). In our study a novel rod shaped liquid crystal having a decyloxy chain has been synthesized and characterized using single-crystal X-ray diffraction study. 4-decylphenyl 4-(benzyloxy)-3- methylbenzoate is the study compound showing monotropic nematic mesophase at 305 K.

The structure of decylphenyl 4-(benzyloxy) -3-methylbenzoate contains one independent molecule in the asymmetric unit. The ring systems and alkyl chain are non coplanar with each other. The dihedral angle between the aromatic rings A–B, B–C and A–C are 66.06 (9)°, 65.21 (8)° and 12.89 (10)° respectively. The alkyl chain and ring C together makes a dihedral angle of 10.73 (12)°. The packing of the molecules is stabilized by C7—H7B···O2 hydrogen bond and Van der Waal's forces(Figure 2).

### Experimental

A mixture of 3-methyl-4-benzyloxybenzoic acid(0.41 mol) and 4-decylphenol(0.41 mol), a catalytic quantity of 4-(*N*,*N*-Dimethylamino) pyridine (DMAP) and dry dichloromethane(10 ml) were stirred for 10 min. To this *N*, *N'*-dicyclohexylcarbodiimide(DCC,0.49 mol) was added and the mixture stirred overnight at room temperature. The precipitated *N*,*N'*-dicyclohexylurea was filtered off; the filterate was diluted with dichloromethane(30 ml) and washed successively with 5% acetic acid(10 ml *x* 2), 5% ice-cold sodium hydroxide solution(10 ml *x* 2) and water(20 ml *x* 3), then dried over anhydrous sodium sulfate.Removal of solvent gave a product which was chromatographed on silica gel using dichloromethane as eluent. Removal of solvent from the eluate afforded a white product which was recrystallized with analytical grade methanol. Yield was about 90%. m.p. 339 K. Spectral data IR (KBr) cm^-1^: 2951 & 2891(CH_2_ aliphatic), 1728(C=O ester),1602(aromatic C=C), 1469(CH aromatic). ^1^H NMR(CDCl_3_): 8.01(d, 2H, Ar—H), 7.43(m, 5H, Ar—H), 7.33(d, 1H, Ar—H),7.20(d, 2H, Ar—H), 7.10(d, 2H, Ar—H), 5.20(s, 2H, Ar—CH_2_—O–), 2.56(t, 2H, Ar—CH_2_–), 2.30(s, 3H, Ar—CH_3_), 1.56(m,2*H*, Ar—CH_2_—CH_2_–), 1.30(m, 14H, aliphatic-CH_2_–),0.9 (t, 3H, –CH_3_). Elemental analysis: Molecular Weight, 458.63 for C_31_ H_38_O_3_requires C 81.18%, H 8.35%. Found, C 80.71% H 8.50%.

### Refinement

All H atoms were positioned at calculated positions with C—H = 0.93Å for aromatic H, 0.97Å for methylene H and 0.96Å for methyl H and refined using a riding model with *U*_iso_(H) =1.5*U*_eq_(C)for methyl H and 1.2*U*_eq_(C) for other.

### Crystal data

**Table:**

C_31_H_38_O_3_	*Z* = 2
*M**_r_* = 458.61	*F*(000) = 496
Triclinic, *P*1	*D*_x_ = 1.138 Mg m^−^^3^
Hall symbol: -P 1	Melting point: 339 K
*a* = 9.3684 (16) Å	Mo *K*α radiation, λ = 0.71073 Å
*b* = 11.168 (2) Å	Cell parameters from 6737 reflections
*c* = 15.204 (3) Å	θ = 1.5–28.5°
α = 68.588 (11)°	µ = 0.07 mm^−^^1^
β = 87.274 (11)°	*T* = 295 K
γ = 65.578 (10)°	Plate, colourless
*V* = 1338.3 (4) Å^3^	0.22 × 0.15 × 0.12 mm

### Data collection

**Table:**

Bruker SMART CCD area-detector diffractometer	6737 independent reflections
Radiation source: fine-focus sealed tube	3840 reflections with *I* > 2σ(*I*)
graphite	*R*_int_ = 0.034
ω and φ scans	θ_max_ = 28.5°, θ_min_ = 1.5°
Absorption correction: ψ scan (*SADABS*; Sheldrick, 2004)	*h* = −12→12
*T*_min_ = 0.987, *T*_max_ = 0.992	*k* = −14→14
23986 measured reflections	*l* = −19→20

### Refinement

**Table:**

Refinement on *F*^2^	Secondary atom site location: difference Fourier map
Least-squares matrix: full	Hydrogen site location: inferred from neighbouring sites
*R*[*F*^2^ > 2σ(*F*^2^)] = 0.053	H-atom parameters constrained
*wR*(*F*^2^) = 0.183	*w* = 1/[σ^2^(*F*_o_^2^) + (0.0834*P*)^2^ + 0.1711*P*] where *P* = (*F*_o_^2^ + 2*F*_c_^2^)/3
*S* = 1.04	(Δ/σ)_max_ < 0.001
6737 reflections	Δρ_max_ = 0.37 e Å^−^^3^
308 parameters	Δρ_min_ = −0.28 e Å^−^^3^
6 restraints	Extinction correction: *SHELXL97* (Sheldrick, 2008), Fc^*^=kFc[1+0.001xFc^2^λ^3^/sin(2θ)]^-1/4^
Primary atom site location: structure-invariant direct methods	Extinction coefficient: 0.009 (2)

### Special details

**Table:**

Geometry. All s.u.'s (except the s.u. in the dihedral angle between two l.s. planes) are estimated using the full covariance matrix. The cell s.u.'s are taken into account individually in the estimation of s.u.'s in distances, angles and torsion angles; correlations between s.u.'s in cell parameters are only used when they are defined by crystal symmetry. An approximate (isotropic) treatment of cell s.u.'s is used for estimating s.u.'s involving l.s. planes.
Refinement. Refinement of *F*^2^ against ALL reflections. The weighted *R*-factor *wR* and goodness of fit *S* are based on *F*^2^, conventional *R*-factors *R* are based on *F*, with *F* set to zero for negative *F*^2^. The threshold expression of *F*^2^ > 2σ(*F*^2^) is used only for calculating *R*-factors(gt) *etc*. and is not relevant to the choice of reflections for refinement. *R*-factors based on *F*^2^ are statistically about twice as large as those based on *F*, and *R*- factors based on ALL data will be even larger.

### Fractional atomic coordinates and isotropic or equivalent isotropic displacement parameters (Å^2^)

**Table:**

	*x*	*y*	*z*	*U*_iso_*/*U*_eq_	
O1	1.29391 (13)	0.71764 (13)	0.39798 (8)	0.0596 (3)	
O2	0.76009 (16)	0.85364 (16)	0.66658 (10)	0.0782 (4)	
O3	0.66987 (13)	0.76081 (12)	0.58579 (8)	0.0587 (3)	
C1	1.7436 (2)	0.6890 (3)	0.21401 (16)	0.0773 (6)	
H1	1.8161	0.6725	0.1710	0.093*	
C2	1.7328 (2)	0.5776 (2)	0.28609 (18)	0.0808 (6)	
H2	1.7987	0.4849	0.2926	0.097*	
C3	1.6246 (2)	0.6016 (2)	0.34940 (15)	0.0734 (5)	
H3	1.6178	0.5249	0.3983	0.088*	
C4	1.52641 (19)	0.73813 (19)	0.34098 (13)	0.0563 (4)	
C5	1.5386 (2)	0.85005 (19)	0.26763 (14)	0.0654 (5)	
H5	1.4728	0.9431	0.2605	0.078*	
C6	1.6476 (2)	0.8246 (2)	0.20518 (15)	0.0770 (6)	
H6	1.6560	0.9006	0.1565	0.092*	
C8	1.16997 (18)	0.74083 (16)	0.45050 (11)	0.0493 (4)	
C7	1.4092 (2)	0.7663 (2)	0.40939 (14)	0.0703 (5)	
H7A	1.4619	0.7163	0.4741	0.084*	
H7B	1.3582	0.8672	0.3967	0.084*	
C9	1.05899 (18)	0.69493 (15)	0.43409 (11)	0.0478 (4)	
C10	0.93050 (18)	0.71552 (16)	0.48486 (11)	0.0490 (4)	
H10	0.8552	0.6864	0.4746	0.059*	
C11	0.91080 (18)	0.77889 (16)	0.55122 (11)	0.0493 (4)	
C12	1.0225 (2)	0.82287 (19)	0.56523 (12)	0.0578 (4)	
H12	1.0102	0.8657	0.6090	0.069*	
C13	1.1519 (2)	0.80452 (19)	0.51555 (12)	0.0589 (4)	
H13	1.2262	0.8347	0.5257	0.071*	
C14	1.0830 (2)	0.6250 (2)	0.36370 (14)	0.0680 (5)	
H14A	1.1784	0.6208	0.3362	0.102*	
H14B	0.9950	0.6789	0.3144	0.102*	
H14C	1.0908	0.5304	0.3955	0.102*	
C15	0.77674 (19)	0.80247 (17)	0.60752 (12)	0.0538 (4)	
C16	0.53999 (18)	0.77559 (18)	0.63921 (11)	0.0512 (4)	
C17	0.5292 (2)	0.65529 (18)	0.70043 (13)	0.0611 (5)	
H17	0.6060	0.5660	0.7067	0.073*	
C18	0.4030 (2)	0.66829 (18)	0.75268 (13)	0.0604 (4)	
H18	0.3958	0.5864	0.7943	0.072*	
C19	0.28666 (19)	0.79952 (17)	0.74503 (11)	0.0501 (4)	
C20	0.30000 (19)	0.91846 (17)	0.68052 (12)	0.0561 (4)	
H20	0.2225	1.0082	0.6727	0.067*	
C21	0.42535 (19)	0.90750 (18)	0.62747 (12)	0.0553 (4)	
H21	0.4318	0.9888	0.5843	0.066*	
C22	0.1539 (2)	0.80822 (18)	0.80631 (13)	0.0608 (4)	
H22A	0.0896	0.7711	0.7865	0.073*	
H22B	0.1997	0.7462	0.8716	0.073*	
C23	0.0474 (2)	0.95323 (19)	0.80478 (13)	0.0612 (5)	
H23A	−0.0087	1.0130	0.7413	0.073*	
H23B	0.1116	0.9952	0.8184	0.073*	
C24	−0.0723 (2)	0.9519 (2)	0.87570 (13)	0.0650 (5)	
H24A	−0.1399	0.9143	0.8601	0.078*	
H24B	−0.0164	0.8883	0.9388	0.078*	
C25	−0.1748 (2)	1.0969 (2)	0.87793 (13)	0.0634 (5)	
H25A	−0.2347	1.1594	0.8157	0.076*	
H25B	−0.1073	1.1365	0.8908	0.076*	
C26	−0.2886 (2)	1.0926 (2)	0.95230 (13)	0.0632 (5)	
H26A	−0.3508	1.0468	0.9419	0.076*	
H26B	−0.2280	1.0347	1.0147	0.076*	
C27	−0.4000 (2)	1.23739 (19)	0.95192 (13)	0.0639 (5)	
H27A	−0.3383	1.2817	0.9654	0.077*	
H27B	−0.4578	1.2970	0.8889	0.077*	
C28	−0.5169 (2)	1.23037 (19)	1.02399 (13)	0.0641 (5)	
H28A	−0.5745	1.1819	1.0119	0.077*	
H28B	−0.4582	1.1731	1.0870	0.077*	
C29	−0.6347 (2)	1.3720 (2)	1.02406 (14)	0.0687 (5)	
H29A	−0.5781	1.4193	1.0389	0.082*	
H29B	−0.6917	1.4310	0.9607	0.082*	
C30	−0.7516 (2)	1.3592 (2)	1.09440 (16)	0.0803 (6)	
H30A	−0.6939	1.2939	1.1567	0.096*	
H30B	−0.8133	1.3183	1.0764	0.096*	
C31	−0.8634 (3)	1.4982 (3)	1.1015 (2)	0.1154 (9)	
H31A	−0.9338	1.4814	1.1475	0.173*	
H31B	−0.9235	1.5629	1.0406	0.173*	
H31C	−0.8039	1.5386	1.1210	0.173*	

### Atomic displacement parameters (Å^2^)

**Table:**

	*U*^11^	*U*^22^	*U*^33^	*U*^12^	*U*^13^	*U*^23^
O1	0.0546 (7)	0.0750 (8)	0.0666 (7)	−0.0390 (6)	0.0209 (6)	−0.0339 (6)
O2	0.0796 (9)	0.1107 (11)	0.0799 (9)	−0.0556 (8)	0.0335 (7)	−0.0593 (9)
O3	0.0524 (7)	0.0696 (7)	0.0669 (7)	−0.0305 (6)	0.0233 (6)	−0.0365 (6)
C1	0.0557 (11)	0.0991 (17)	0.0911 (15)	−0.0335 (11)	0.0254 (10)	−0.0522 (14)
C2	0.0535 (11)	0.0668 (13)	0.1176 (18)	−0.0144 (10)	0.0078 (12)	−0.0426 (13)
C3	0.0661 (12)	0.0579 (11)	0.0869 (14)	−0.0293 (10)	0.0077 (11)	−0.0141 (10)
C4	0.0497 (9)	0.0661 (11)	0.0632 (10)	−0.0336 (8)	0.0118 (8)	−0.0257 (9)
C5	0.0630 (11)	0.0566 (10)	0.0798 (12)	−0.0285 (9)	0.0197 (10)	−0.0272 (10)
C6	0.0774 (13)	0.0784 (14)	0.0781 (13)	−0.0423 (11)	0.0282 (11)	−0.0246 (11)
C8	0.0475 (8)	0.0491 (9)	0.0492 (9)	−0.0228 (7)	0.0098 (7)	−0.0143 (7)
C7	0.0669 (11)	0.0982 (15)	0.0767 (12)	−0.0548 (11)	0.0256 (10)	−0.0456 (11)
C9	0.0482 (8)	0.0435 (8)	0.0505 (9)	−0.0206 (7)	0.0084 (7)	−0.0156 (7)
C10	0.0464 (8)	0.0461 (8)	0.0543 (9)	−0.0219 (7)	0.0086 (7)	−0.0167 (7)
C11	0.0470 (8)	0.0468 (9)	0.0483 (9)	−0.0183 (7)	0.0071 (7)	−0.0138 (7)
C12	0.0614 (10)	0.0672 (11)	0.0534 (9)	−0.0313 (9)	0.0119 (8)	−0.0283 (8)
C13	0.0579 (10)	0.0720 (11)	0.0612 (10)	−0.0376 (9)	0.0123 (8)	−0.0297 (9)
C14	0.0688 (11)	0.0793 (12)	0.0822 (13)	−0.0439 (10)	0.0274 (10)	−0.0466 (11)
C15	0.0533 (9)	0.0544 (10)	0.0533 (9)	−0.0239 (8)	0.0114 (8)	−0.0193 (8)
C16	0.0487 (9)	0.0589 (10)	0.0524 (9)	−0.0245 (8)	0.0147 (7)	−0.0272 (8)
C17	0.0562 (10)	0.0496 (10)	0.0747 (12)	−0.0189 (8)	0.0180 (9)	−0.0260 (9)
C18	0.0635 (11)	0.0488 (9)	0.0668 (11)	−0.0254 (8)	0.0186 (9)	−0.0191 (8)
C19	0.0526 (9)	0.0529 (9)	0.0463 (8)	−0.0235 (8)	0.0113 (7)	−0.0200 (7)
C20	0.0555 (10)	0.0482 (9)	0.0569 (10)	−0.0177 (8)	0.0141 (8)	−0.0176 (8)
C21	0.0565 (10)	0.0521 (9)	0.0539 (9)	−0.0239 (8)	0.0157 (8)	−0.0165 (8)
C22	0.0629 (10)	0.0609 (10)	0.0570 (10)	−0.0274 (9)	0.0208 (8)	−0.0212 (8)
C23	0.0589 (10)	0.0642 (11)	0.0607 (10)	−0.0265 (9)	0.0209 (8)	−0.0252 (9)
C24	0.0634 (11)	0.0686 (12)	0.0645 (11)	−0.0301 (9)	0.0266 (9)	−0.0268 (9)
C25	0.0613 (10)	0.0698 (12)	0.0623 (10)	−0.0303 (9)	0.0229 (9)	−0.0275 (9)
C26	0.0605 (10)	0.0658 (11)	0.0616 (10)	−0.0261 (9)	0.0214 (9)	−0.0249 (9)
C27	0.0634 (11)	0.0632 (11)	0.0679 (11)	−0.0285 (9)	0.0213 (9)	−0.0275 (9)
C28	0.0654 (11)	0.0616 (11)	0.0658 (11)	−0.0271 (9)	0.0217 (9)	−0.0261 (9)
C29	0.0682 (12)	0.0637 (11)	0.0731 (12)	−0.0270 (9)	0.0228 (10)	−0.0279 (10)
C30	0.0715 (12)	0.0821 (14)	0.0890 (14)	−0.0307 (11)	0.0319 (11)	−0.0393 (12)
C31	0.0906 (17)	0.108 (2)	0.138 (2)	−0.0207 (15)	0.0451 (16)	−0.0656 (18)

### Geometric parameters (Å, °)

**Table:**

O1—C8	1.3664 (18)	C18—C19	1.384 (2)
O1—C7	1.436 (2)	C18—H18	0.9300
O2—C15	1.2021 (19)	C19—C20	1.384 (2)
O3—C15	1.363 (2)	C19—C22	1.510 (2)
O3—C16	1.4161 (18)	C20—C21	1.381 (2)
C1—C6	1.362 (3)	C20—H20	0.9300
C1—C2	1.363 (3)	C21—H21	0.9300
C1—H1	0.9300	C22—C23	1.502 (2)
C2—C3	1.377 (3)	C22—H22A	0.9700
C2—H2	0.9300	C22—H22B	0.9700
C3—C4	1.377 (3)	C23—C24	1.519 (2)
C3—H3	0.9300	C23—H23A	0.9700
C4—C5	1.381 (2)	C23—H23B	0.9700
C4—C7	1.494 (2)	C24—C25	1.515 (2)
C5—C6	1.375 (3)	C24—H24A	0.9700
C5—H5	0.9300	C24—H24B	0.9700
C6—H6	0.9300	C25—C26	1.518 (2)
C8—C13	1.382 (2)	C25—H25A	0.9700
C8—C9	1.402 (2)	C25—H25B	0.9700
C7—H7A	0.9700	C26—C27	1.517 (2)
C7—H7B	0.9700	C26—H26A	0.9700
C9—C10	1.382 (2)	C26—H26B	0.9700
C9—C14	1.500 (2)	C27—C28	1.517 (2)
C10—C11	1.396 (2)	C27—H27A	0.9700
C10—H10	0.9300	C27—H27B	0.9700
C11—C12	1.380 (2)	C28—C29	1.509 (2)
C11—C15	1.472 (2)	C28—H28A	0.9700
C12—C13	1.380 (2)	C28—H28B	0.9700
C12—H12	0.9300	C29—C30	1.507 (2)
C13—H13	0.9300	C29—H29A	0.9700
C14—H14A	0.9600	C29—H29B	0.9700
C14—H14B	0.9600	C30—C31	1.512 (3)
C14—H14C	0.9600	C30—H30A	0.9700
C16—C17	1.368 (2)	C30—H30B	0.9700
C16—C21	1.369 (2)	C31—H31A	0.9600
C17—C18	1.380 (2)	C31—H31B	0.9600
C17—H17	0.9300	C31—H31C	0.9600
			
C8—O1—C7	117.75 (13)	C21—C20—H20	119.1
C15—O3—C16	116.52 (12)	C19—C20—H20	119.1
C6—C1—C2	119.72 (19)	C16—C21—C20	119.21 (15)
C6—C1—H1	120.1	C16—C21—H21	120.4
C2—C1—H1	120.1	C20—C21—H21	120.4
C1—C2—C3	120.29 (19)	C23—C22—C19	116.47 (14)
C1—C2—H2	119.9	C23—C22—H22A	108.2
C3—C2—H2	119.9	C19—C22—H22A	108.2
C2—C3—C4	120.56 (18)	C23—C22—H22B	108.2
C2—C3—H3	119.7	C19—C22—H22B	108.2
C4—C3—H3	119.7	H22A—C22—H22B	107.3
C3—C4—C5	118.56 (16)	C22—C23—C24	113.59 (14)
C3—C4—C7	121.33 (17)	C22—C23—H23A	108.8
C5—C4—C7	120.12 (17)	C24—C23—H23A	108.8
C6—C5—C4	120.29 (17)	C22—C23—H23B	108.8
C6—C5—H5	119.9	C24—C23—H23B	108.8
C4—C5—H5	119.9	H23A—C23—H23B	107.7
C1—C6—C5	120.58 (19)	C25—C24—C23	113.85 (15)
C1—C6—H6	119.7	C25—C24—H24A	108.8
C5—C6—H6	119.7	C23—C24—H24A	108.8
O1—C8—C13	123.80 (14)	C25—C24—H24B	108.8
O1—C8—C9	115.06 (14)	C23—C24—H24B	108.8
C13—C8—C9	121.13 (14)	H24A—C24—H24B	107.7
O1—C7—C4	108.23 (14)	C24—C25—C26	113.16 (15)
O1—C7—H7A	110.1	C24—C25—H25A	108.9
C4—C7—H7A	110.1	C26—C25—H25A	108.9
O1—C7—H7B	110.1	C24—C25—H25B	108.9
C4—C7—H7B	110.1	C26—C25—H25B	108.9
H7A—C7—H7B	108.4	H25A—C25—H25B	107.8
C10—C9—C8	117.95 (14)	C27—C26—C25	114.33 (15)
C10—C9—C14	122.26 (14)	C27—C26—H26A	108.7
C8—C9—C14	119.78 (14)	C25—C26—H26A	108.7
C9—C10—C11	121.69 (15)	C27—C26—H26B	108.7
C9—C10—H10	119.2	C25—C26—H26B	108.7
C11—C10—H10	119.2	H26A—C26—H26B	107.6
C12—C11—C10	118.62 (14)	C28—C27—C26	113.14 (15)
C12—C11—C15	117.55 (14)	C28—C27—H27A	109.0
C10—C11—C15	123.83 (15)	C26—C27—H27A	109.0
C13—C12—C11	121.27 (15)	C28—C27—H27B	109.0
C13—C12—H12	119.4	C26—C27—H27B	109.0
C11—C12—H12	119.4	H27A—C27—H27B	107.8
C12—C13—C8	119.34 (16)	C29—C28—C27	115.36 (16)
C12—C13—H13	120.3	C29—C28—H28A	108.4
C8—C13—H13	120.3	C27—C28—H28A	108.4
C9—C14—H14A	109.5	C29—C28—H28B	108.4
C9—C14—H14B	109.5	C27—C28—H28B	108.4
H14A—C14—H14B	109.5	H28A—C28—H28B	107.5
C9—C14—H14C	109.5	C30—C29—C28	113.17 (16)
H14A—C14—H14C	109.5	C30—C29—H29A	108.9
H14B—C14—H14C	109.5	C28—C29—H29A	108.9
O2—C15—O3	122.27 (15)	C30—C29—H29B	108.9
O2—C15—C11	124.65 (16)	C28—C29—H29B	108.9
O3—C15—C11	113.08 (14)	H29A—C29—H29B	107.8
C17—C16—C21	120.86 (14)	C29—C30—C31	114.4 (2)
C17—C16—O3	118.55 (14)	C29—C30—H30A	108.7
C21—C16—O3	120.56 (14)	C31—C30—H30A	108.7
C16—C17—C18	119.10 (15)	C29—C30—H30B	108.7
C16—C17—H17	120.5	C31—C30—H30B	108.7
C18—C17—H17	120.5	H30A—C30—H30B	107.6
C17—C18—C19	121.97 (16)	C30—C31—H31A	109.5
C17—C18—H18	119.0	C30—C31—H31B	109.5
C19—C18—H18	119.0	H31A—C31—H31B	109.5
C18—C19—C20	117.03 (14)	C30—C31—H31C	109.5
C18—C19—C22	119.99 (15)	H31A—C31—H31C	109.5
C20—C19—C22	122.97 (14)	H31B—C31—H31C	109.5
C21—C20—C19	121.78 (15)		
			
C6—C1—C2—C3	0.5 (3)	C16—O3—C15—C11	177.94 (13)
C1—C2—C3—C4	−0.2 (3)	C12—C11—C15—O2	−2.2 (3)
C2—C3—C4—C5	0.2 (3)	C10—C11—C15—O2	178.07 (17)
C2—C3—C4—C7	−179.54 (17)	C12—C11—C15—O3	177.47 (14)
C3—C4—C5—C6	−0.5 (3)	C10—C11—C15—O3	−2.3 (2)
C7—C4—C5—C6	179.25 (17)	C15—O3—C16—C17	−112.35 (17)
C2—C1—C6—C5	−0.8 (3)	C15—O3—C16—C21	69.6 (2)
C4—C5—C6—C1	0.8 (3)	C21—C16—C17—C18	−1.8 (3)
C7—O1—C8—C13	−1.9 (2)	O3—C16—C17—C18	−179.82 (15)
C7—O1—C8—C9	178.12 (15)	C16—C17—C18—C19	0.0 (3)
C8—O1—C7—C4	−176.37 (14)	C17—C18—C19—C20	1.6 (3)
C3—C4—C7—O1	−65.5 (2)	C17—C18—C19—C22	−177.81 (16)
C5—C4—C7—O1	114.78 (18)	C18—C19—C20—C21	−1.4 (2)
O1—C8—C9—C10	−179.88 (13)	C22—C19—C20—C21	177.94 (16)
C13—C8—C9—C10	0.1 (2)	C17—C16—C21—C20	2.0 (3)
O1—C8—C9—C14	0.6 (2)	O3—C16—C21—C20	179.92 (14)
C13—C8—C9—C14	−179.41 (16)	C19—C20—C21—C16	−0.3 (3)
C8—C9—C10—C11	−0.5 (2)	C18—C19—C22—C23	170.77 (16)
C14—C9—C10—C11	179.02 (16)	C20—C19—C22—C23	−8.6 (3)
C9—C10—C11—C12	0.6 (2)	C19—C22—C23—C24	−173.87 (15)
C9—C10—C11—C15	−179.63 (14)	C22—C23—C24—C25	177.16 (16)
C10—C11—C12—C13	−0.4 (3)	C23—C24—C25—C26	−177.39 (15)
C15—C11—C12—C13	179.87 (15)	C24—C25—C26—C27	−176.29 (16)
C11—C12—C13—C8	0.0 (3)	C25—C26—C27—C28	177.49 (16)
O1—C8—C13—C12	−179.87 (15)	C26—C27—C28—C29	−177.76 (16)
C9—C8—C13—C12	0.1 (3)	C27—C28—C29—C30	177.89 (17)
C16—O3—C15—O2	−2.4 (2)	C28—C29—C30—C31	175.6 (2)

### Hydrogen-bond geometry (Å, °)

**Table:**

*D*—H···*A*	*D*—H	H···*A*	*D*···*A*	*D*—H···*A*
C10—H10···O3	0.93	2.47	2.786 (2)	100
C14—H14A···O1	0.96	2.24	2.726 (3)	110
